# Left Ventricular Apical Thrombus in Apical Hypertrophic Cardiomyopathy Without Aneurysm or Arrhythmia: A Case Report

**DOI:** 10.1002/ccd.70265

**Published:** 2025-10-13

**Authors:** Ibrahim Antoun, Jeffrey Khoo, Sanjay S. Bhandari

**Affiliations:** ^1^ Department of Cardiology University Hospitals of Leicester NHS Turst Leicester UK; ^2^ Department of Cardiovascular Sciences Leicester UK

**Keywords:** apical hypertrophic cardiomyopathy, cardiac magnetic resonance, case report, echocardiography, thrombus

## Abstract

Apical hypertrophic cardiomyopathy (ApHCM) is a rare variant of hypertrophic cardiomyopathy, typically associated with a benign course. However, complications such as ventricular arrhythmias, apical aneurysms, and thrombus formation may occur. Left ventricular (LV) thrombus is an unusual finding in ApHCM, especially in patients with preserved systolic function and normal sinus rhythm. A 54‐year‐old male with a history of pulmonary embolism and ApHCM was under routine surveillance. He remained asymptomatic with a normal sinus rhythm. Transthoracic echocardiography (TTE) identified an echogenic mass in the LV apex. Cardiac magnetic resonance imaging (CMR) confirmed severe apical hypertrophy, preserved LV systolic function, and a large apical mass measuring 24 × 19 mm. The mass showed no contrast uptake on early or late gadolinium enhancement sequences, consistent with thrombus. Native T1 mapping was mildly elevated, suggesting diffuse interstitial fibrosis, and focal non‐ischaemic replacement fibrosis was noted. There was no evidence of apical aneurysm or mid‐ventricular obstruction. The patient was anticoagulated with warfarin and remains under close follow‐up. This case represents a rare occurrence of a large LV thrombus in ApHCM without associated apical aneurysm or impaired LV function. The findings suggest that regional fibrosis and altered apical flow dynamics may contribute to thrombus formation even in hypercontractile ventricles. Multimodal imaging, particularly CMR, is essential for accurate diagnosis and risk assessment. Clinicians should maintain vigilance for thrombotic complications in ApHCM, even in the absence of classical risk factors, as subtle fibrosis or flow abnormalities may predispose to thrombus formation.

## 1 Introduction

Cardiovascular disease is increasing in prevalence, and its management is challenging, especially in the developing world [1, 2, 3, 4]. Apical hypertrophic cardiomyopathy (ApHCM) is a distinct morphological variant of hypertrophic cardiomyopathy (HCM), predominantly affecting the left ventricular (LV) apex [5]. While the overall prognosis of ApHCM is often favorable, complications such as apical aneurysm formation, ventricular arrhythmias, and thromboembolic events can occur, particularly in the presence of apical myocardial thinning or cavity dilation. LV thrombus is an uncommon finding in HCM and is typically associated with impaired systolic function, apical aneurysm, or the presence of atrial fibrillation [6]. The development of a large LV thrombus in a patient with preserved LV systolic function and normal sinus rhythm is rare and raises important questions about underlying pathophysiological mechanisms. We report a rare case of a large LV thrombus in a patient with ApHCM, preserved ejection fraction, and no evidence of apical aneurysm or atrial arrhythmias. This case highlights the importance of multimodal imaging and vigilant follow‐up in this population.

## 2 Case Presentation

**Figure 1:**
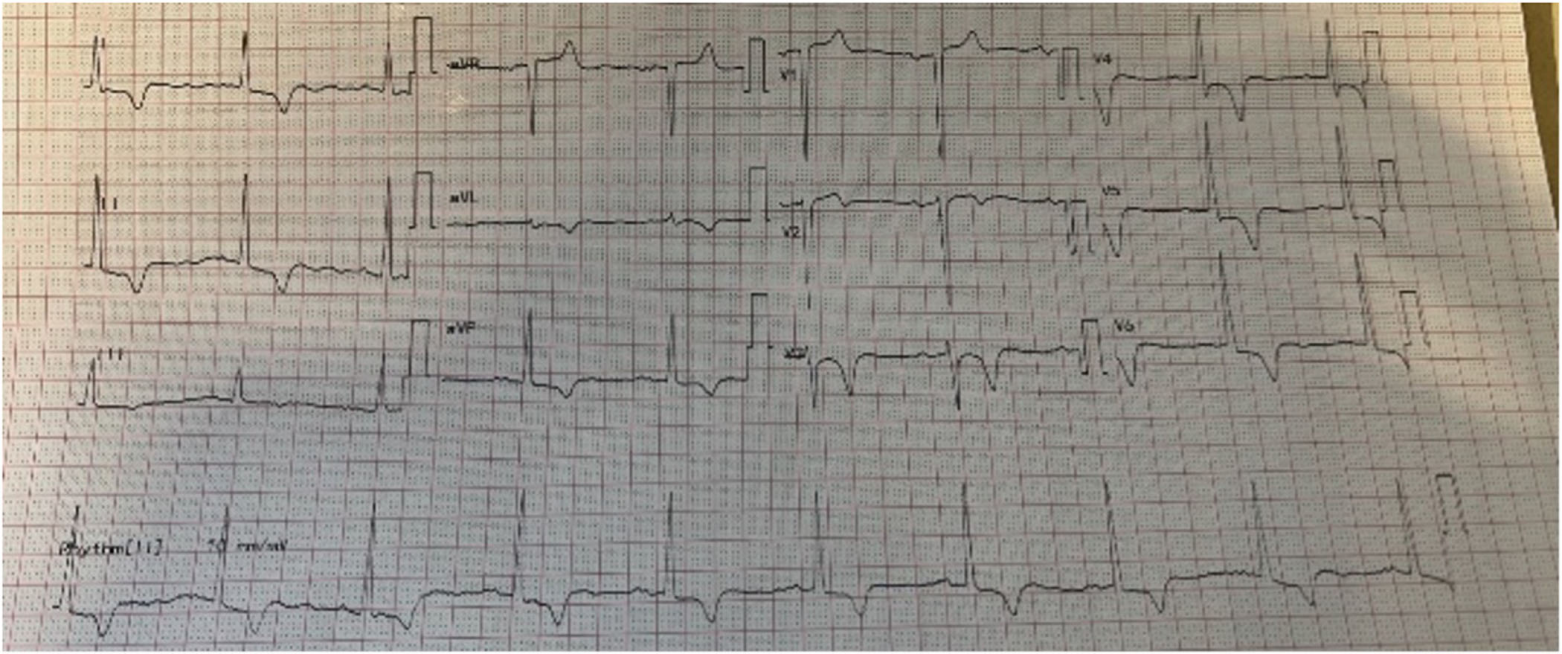
The patient's 12‐lead electrocardiogram (ECG) demonstrating features suggestive of apical hypertrophic cardiomyopathy. There is deep symmetrical T‐wave inversion in the precordial leads (V3–V6) and inferior leads (II, III, aVF), a hallmark of apical hypertrophy. The QRS duration is normal, and there is no evidence of pathological Q waves or conduction abnormalities. The patient was in sinus rhythm at the time of recording. [Color figure can be viewed at wileyonlinelibrary.com]

**Figure 2:**
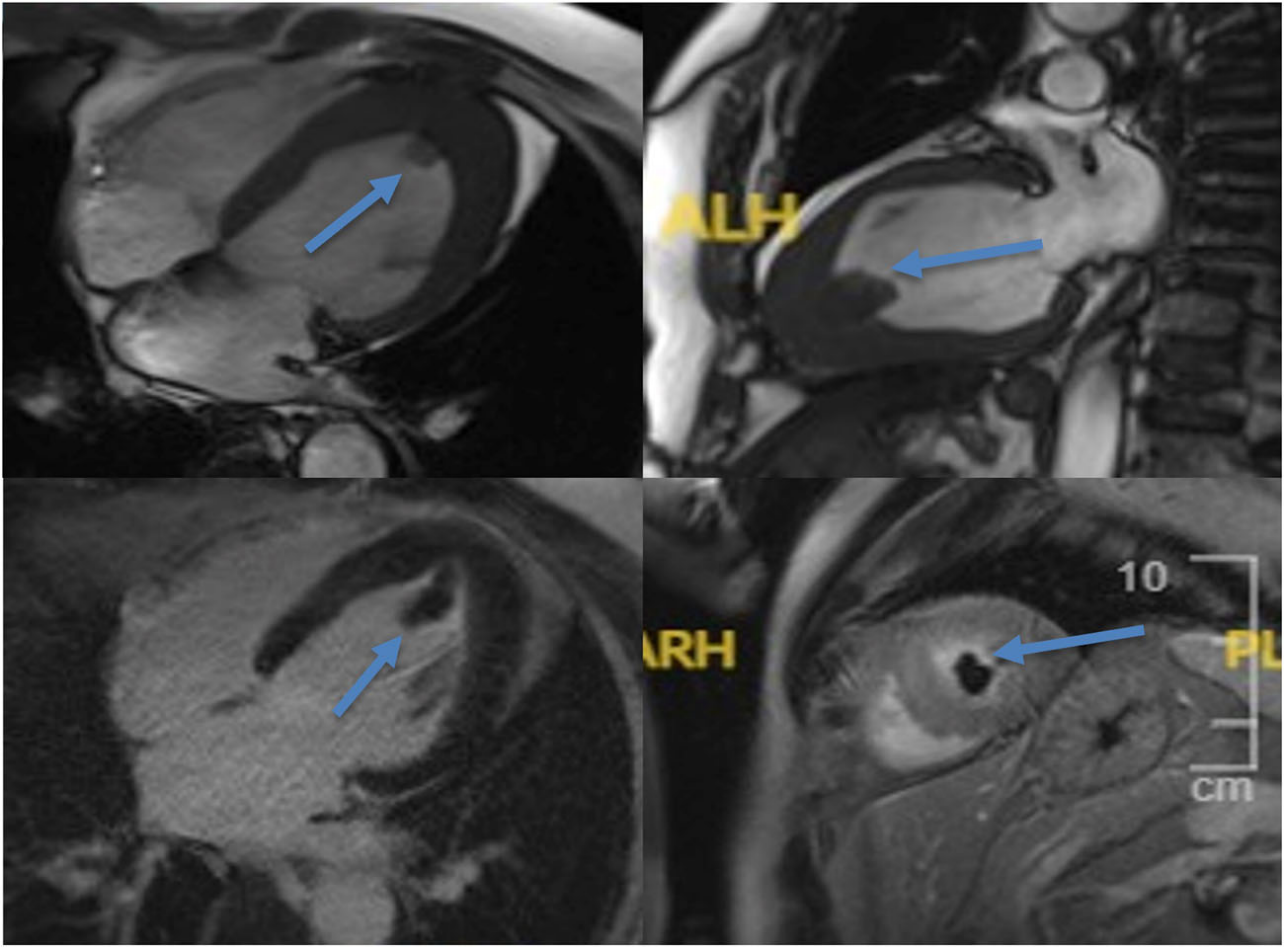
Cardiac magnetic resonance imaging demonstrating a large left ventricular (LV) apical thrombus in a patient with apical hypertrophic cardiomyopathy (ApHCM). (Top left and top right): Steady‐state free precession (SSFP) cine images in long‐axis views show a well‐demarcated apical mass (arrows) within a hypertrophied LV apex. (Bottom left): Early post‐contrast inversion recovery image demonstrates lack of enhancement of the apical mass, consistent with thrombus (arrow). (Bottom right): Late gadolinium enhancement (LGE) image further confirms the non‐enhancing nature of the apical thrombus (arrow), with surrounding myocardial enhancement indicating apical fibrosis. No apical aneurysm is evident. [Color figure can be viewed at wileyonlinelibrary.com]

A 54‐year‐old male with a history of pulmonary embolism and ApHCM was under routine annual surveillance. He remained asymptomatic, and his last 12‐lead electrocardiogram (ECG) showed a normal sinus rhythm with deep symmetrical T‐wave inversion in the precordial leads (V3–V6) and inferior leads (II, III, aVF), a hallmark of ApHCM (Figure 1). A transthoracic echocardiogram (TTE) unexpectedly revealed an echogenic mass in the LV apex. LV systolic function was preserved, with no regional wall motion abnormalities (RWMA). Cardiac magnetic resonance imaging (CMR) was performed to further evaluate the nature of the mass. CMR demonstrated severe apical LV hypertrophy, preserved global LV systolic function, and a large, well‐defined apical mass measuring 24 × 19 mm. There was no evidence of an apical aneurysm. The mass showed no contrast uptake on both early and late gadolinium enhancement (LGE) sequences, consistent with a thrombus (Figure 2). Native T1 mapping revealed mildly elevated values (1300–1400 ms), suggesting mild diffuse interstitial fibrosis. LGE additionally identified focal, non‐ischemic replacement fibrosis in the apical anterior wall.

A thrombophilia screen was performed following his initial pulmonary embolism and again after the incidental finding of LV thrombus. Both screenings were negative for inherited or acquired thrombophilic disorders, including Factor V Leiden, prothrombin gene mutation, antithrombin III, protein C and S deficiencies, and antiphospholipid antibodies. No family history of venous or arterial thromboembolism was reported. The patient was not on hormone therapy or immobilized.

TTE confirmed preserved biventricular systolic function and no RWMA. The apical views revealed a homogeneous, echogenic mass within the apex. Images and cine clips are available in the supporting material.

The patient was commenced on oral anticoagulation with warfarin. On interval transthoracic echocardiographic follow‐up, the apical thrombus remained visible but stable in size, with no evidence of embolisation or increase in mobility. The patient remains clinically asymptomatic and is continuing long‐term anticoagulation.

## 3 Discussion

LV thrombus in HCM is an uncommon but clinically significant complication. Most commonly, LV thrombus is associated with ischemic cardiomyopathy, apical aneurysms, or significant systolic dysfunction. In HCM, particularly the apical and mid‐ventricular variants, thrombus formation has been reported but remains rare, especially in the absence of overt apical aneurysm, arrhythmia, or impaired contractility [7].

ApHCM is characterized by asymmetric hypertrophy confined to the apex of the left ventricle [8]. While often benign in presentation, ApHCM can lead to complications such as apical aneurysms, mid‐cavity obstruction, myocardial fibrosis, and arrhythmia [9]. These structural and functional abnormalities can predispose patients to thrombus formation through blood stasis, endothelial injury, or hypercoagulability—components of Virchow's triad [10].

Previous case reports by Kaku and Tezuka et al. describe LV thrombus in the context of mid‐ventricular obstruction, with some patients also demonstrating apical aneurysm or akinetic segments [11, 12]. Fighali et al. suggested that mid‐ventricular obstruction may result in progressive myocardial dysfunction and dilatation of the apical chamber, even in the absence of a discrete aneurysm [13]. Furthermore, Hamada et al. also reported that altered intraventricular flow patterns and abnormal pressure gradients in mid‐ventricular obstruction could promote blood stasis and thrombus formation [6].

In contrast to previous cases, our patient had preserved LV function, no aneurysm, and was in sinus rhythm. The development of a large thrombus in this context is multifactorial. First, focal and diffuse fibrosis (identified by LGE and elevated native T1 values) may contribute to local contractile abnormalities and disturbed blood flow. Second, although the patient was in sinus rhythm, reduced diastolic filling and apical cavity obliteration during systole might result in sluggish flow and blood stasis. Third, despite preserved global function, regional kinetic abnormalities may exist but remain undetectable on standard imaging. Finally, his history of pulmonary embolism raises suspicion for a hypercoagulable state, though no thrombophilia was found.

While this presentation may not be exceedingly rare, it remains clinically significant due to the asymptomatic nature, preserved systolic function, and the absence of overt apical aneurysm—all of which can lead to missed diagnosis if imaging is not thorough. This case highlights the importance of vigilance and the value of CMR in this population.

The pathophysiology underlying LV thrombus in this setting remains incompletely understood. Hayashi et al. proposed that myocardial damage and necrosis may reduce active suction during diastole, particularly in fibrotic or scarred myocardium, impairing LV filling and creating conditions conducive to blood pooling and thrombogenesis [7, 14, 15]. In apical HCM, even without an aneurysm, such fibrotic regions may subtly alter apical mechanics, contributing to thrombus formation. Although global systolic function and sinus rhythm were preserved, several ApHCM‐specific mechanisms can favor mural thrombosis. First, apical cavity obliteration during systole may create low‐shear zones in diastole, resulting in impaired washout, a physiology observed in apical and mid‐ventricular HCM and linked to downstream apical remodeling, even before aneurysm formation [16]. Second, coronary microvascular dysfunction, a cardinal feature of HCM and particularly evident in apical HCM, produces regional ischemia and reduced diastolic suction that can promote stasis [17]. Third, diffuse interstitial fibrosis, as reflected by elevated native T1, can subtly impair regional relaxation without generating a discrete endocardial LGE pattern [18]. Finally, prothrombotic changes, including enhanced thrombin generation and platelet activation, have been reported in HCM and may lower the threshold for mural thrombus formation [19]. In this context, the non‐enhancing apical mass on early and late gadolinium sequences is characteristic of thrombus. At the same time, the absence of endocardial LGE does not exclude diffuse fibrosis or microvascular ischemia as upstream contributors.

This case highlights the importance of routine and detailed surveillance in patients with apical HCM. TTE may detect apical masses, but tissue characterization with CMR is critical for differentiating thrombus from tumor or artefact. While anticoagulation is the mainstay of treatment, its timing and duration in HCM patients with thrombus but preserved systolic function remain uncertain and warrant further research [20]. Given the potentially devastating consequences of embolic events, clinicians should maintain a high index of suspicion for LV thrombus in HCM patients, even those with preserved LV function and no arrhythmia, particularly if focal fibrosis or structural abnormalities are present. This case underscores the importance of multimodal imaging and the need to better understand thrombotic risk in this patient population.

## Consent

The authors confirm that the patient gave informed consent to publish this case report.

## Conflicts of Interest

The authors declare no conflicts of interest.

## Data Availability

The data that support the findings of this study are available from the corresponding author upon reasonable request.

## References

[1] I. Antoun , A. Alkhayer , M. Aljabal , et al., “Thirty‐Day Unplanned Readmissions Following Hospitalization for Atrial Fibrillation in a Tertiary Syrian Center: A Real‐World Observational Cohort Study,” Heart Rhythm O2 5, no. 12 (2024): 854–859. 39803619 10.1016/j.hroo.2024.05.010 PMC11721721

[2] I. Antoun , M. Aljabal , A. Alkhayer , et al., “Atrial Fibrillation Inpatient Management Patterns and Clinical Outcomes During the Conflict in Syria: An Observational Cohort Study,” Perfusion 40, no. 3 (2024): 02676591241259140. 10.1177/02676591241259140 PMC11951463 38830625

[3] I. Antoun , A. Alkhayer , M. Aljabal , et al., “Incidence, Outcomes, and Predictors of New Heart Failure in Syrian Conflict‐Affected Population Following Hospitalization for Atrial Fibrillation: A Retrospective Cohort Study,” Perfusion 40, no. 5 (2024): 02676591241283883. 10.1177/02676591241283883 PMC12202832 39255054

[4] I. Antoun , A. Alkhayer , A. Alkhayer , et al., “Six‐Month Emergent Readmissions Following Hospitalization for Atrial Fibrillation Amid the Syrian Conflict: A Real‐World Observational Cohort Study,” Journal of Cardiovascular Electrophysiology 36 (2025): 582–588. 39803787 10.1111/jce.16579 PMC11903379

[5] R. K. Hughes , K. D. Knott , J. Malcolmson , et al., “Apical Hypertrophic Cardiomyopathy: The Variant Less Known,” Journal of the American Heart Association 9, no. 5 (2020): e015294. 32106746 10.1161/JAHA.119.015294 PMC7335568

[6] M. Hamada , “Left Ventricular Thrombus in Hypertrophic Cardiomyopathy,” Internal Medicine 58, no. 4 (2019): 465–467. 30333417 10.2169/internalmedicine.1646-18 PMC6421158

[7] C. P. McCarthy , S. Murphy , R. V. Venkateswaran , et al., “Left Ventricular Thrombus,” Journal of the American College of Cardiology 73, no. 15 (2019): 2007–2009. 30846340 10.1016/j.jacc.2019.01.031

[8] I. Antoun and S. S. Bhandari , “Not All That Thickens is Hypertrophic Cardiomyopathy: A Case Report of Unusual Takotsubo Cardiomyopathy,” European Heart Journal—Case Reports 9, no. 9 (2025): ytaf427. 41141988 10.1093/ehjcr/ytaf427 PMC12548529

[9] M. F. Jan , M. C. Todaro , L. Oreto , and A. J. Tajik , “Apical Hypertrophic Cardiomyopathy: Present Status,” International Journal of Cardiology 222 (2016): 745–759. 27521551 10.1016/j.ijcard.2016.07.154

[10] A. Kushner , W. P. West , M. Z. K. Suheb , and L. S. Pillarisetty , “Virchow triad.” StatPearls [Internet] (StatPearls Publishing, 2022). 30969519

[11] B. Kaku , “Intra‐Cardiac Thrombus Resolution After Anti‐Coagulation Therapy With Dabigatran in a Patient With Mid‐Ventricular Obstructive Hypertrophic Cardiomyopathy: A Case Report,” Journal of medical case reports 7 (2013): 238. 24103078 10.1186/1752-1947-7-238 PMC3851832

[12] A. Tezuka , K. Higo , Y. Nakamukae , et al., “Bisoprolol Successfully Improved the Intraventricular Pressure Gradient in a Patient With Midventricular Obstructive Hypertrophic Cardiomyopathy With an Apex Aneurysm Due to Apical Myocardial Damage,” Internal Medicine 58, no. 4 (2019): 535–539. 30333393 10.2169/internalmedicine.0997-18 PMC6421145

[13] S. Fighali , Z. Krajcer , S. Edelman , and R. D. Leachman , “Progression of Hypertrophic Cardiomyopathy into a Hypokinetic Left Ventricle: Higher Incidence in Patients With Midventricular Obstruction,” Journal of the American College of Cardiology 9, no. 2 (1987): 288–294. 3805517 10.1016/s0735-1097(87)80377-7

[14] M. Massussi , A. Scotti , G. Y. H. Lip , and R. Proietti , “Left Ventricular Thrombosis: New Perspectives on an Old Problem,” European Heart Journal‐Cardiovascular Pharmacotherapy 7, no. 2 (2021): 158–167. 32569361 10.1093/ehjcvp/pvaa066

[15] P. J. Stokman , C. S. Nandra , and R. W. Asinger , “Left Ventricular Thrombus,” Current Treatment Options in Cardiovascular Medicine 3, no. 6 (2001): 515–521. 11696271 10.1007/s11936-001-0025-6

[16] Y. Sato , N. Matsumoto , S. Matsuo , et al., “Mid‐Ventricular Obstructive Hypertrophic Cardiomyopathy Associated With an Apical Aneurysm: Evaluation of Possible Causes of Aneurysm Formation,” Yonsei Medical Journal 48, no. 5 (2007): 879–882. 17963350 10.3349/ymj.2007.48.5.879 PMC2628158

[17] F. Pelliccia , F. Cecchi , I. Olivotto , and P. Camici , “Microvascular Dysfunction in Hypertrophic Cardiomyopathy,” Journal of Clinical Medicine 11, no. 21 (2022): 6560. 36362787 10.3390/jcm11216560 PMC9658510

[18] J. Gleditsch , Ø. Jervan , M. Tavoly , et al., “Association Between Myocardial Fibrosis, as Assessed With Cardiac Magnetic Resonance T1 Mapping, and Persistent Dyspnea After Pulmonary Embolism,” IJC Heart & Vasculature 38 (2022): 100935. 35005213 10.1016/j.ijcha.2021.100935 PMC8717259

[19] Q. Fan , Z. Lu , Y. Wang , et al., “Association Between Postoperative Nadir Platelet Count and Postoperative Cardiovascular Complications Following Septal Myectomy in Patients With Hypertrophic Cardiomyopathy: A Retrospective Cohort Study,” BMC Cardiovascular Disorders 24, no. 1 (2024): 57. 38238666 10.1186/s12872-024-03724-2 PMC10795313

[20] P. Goyal and J. W. Weinsaft , “Cardiovascular Magnetic Resonance Imaging for Assessment of Cardiac Thrombus,” Methodist DeBakey Cardiovascular Journal 9, no. 3 (2013): 132. 24066195 10.14797/mdcj-9-3-132 PMC3782319

